# Gastric Signet Ring Cell Adenocarcinoma Presenting as Intermittent Volvulus of Small Bowel: A Case Presentation and Review of the Literature

**DOI:** 10.7759/cureus.23228

**Published:** 2022-03-16

**Authors:** Anum Aqsa, Sami Droubi, Hassan Al-Moussawi, Gloria Lan, Sherif Andrawes

**Affiliations:** 1 Internal Medicine, Staten Island University Hospital - Northwell Health, New York, USA; 2 Gastroenterology and Hepatology, Staten Island University Hospital - Northwell Health, New York, USA; 3 Gastroenterology, Staten Island University Hospital - Northwell Health, New York, USA

**Keywords:** rare cancers, signet-ring cell carcinoma, gastric adenocarcinoma, small bowel volvulus, jejunal volvulus

## Abstract

Signet ring cell carcinoma (SRCC) is an uncommon and poorly differentiated tumor. It arises mostly in the gastrointestinal tract. The incidence of gastric SRCC has increased in the past few years. Volvulus is the twisting of the bowel around its mesentery. It is classified as either primary or secondary. It is relatively common in the cecum and sigmoid colon. Volvulus of other parts of the gastrointestinal tract is relatively rare. Herein, we present a case of small bowel volvulus (SBV) secondary to advanced gastric SRCC with peritoneal carcinomatosis. The patient had presented with nausea and vomiting. Initial computed tomography (CT) scan of abdomen unveiled jejunal volvulus. SBV resolved spontaneously on a repeat CT scan. Enteroscopy with histopathology confirmed the diagnosis of gastric SRCC, which turned to be metastatic to peritoneum on laparoscopy. We believe our case is unique due to the rarity of advanced gastric SRCC presenting as secondary jejunal SBV without appreciated gastric mass on imaging.

## Introduction

Signet ring cell carcinoma (SRCC) is a poorly differentiated adenocarcinoma. It arises in the stomach in 90% of cases [1]. Less frequent sites include the gallbladder, pancreas, breast, colon, and urinary bladder. Histologically it is characterized by the presence of signet ring cells in which excessive intracytoplasmic mucin pushes the nucleus to the periphery.

Gastric signet ring cell adenocarcinoma (SRCA/SRCC) belongs to the diffuse type (lack of glandular growth, poorly differentiated) of gastric cancer (GC) according to Lauren classification and is poorly cohesive as per WHO classification. Its incidence has increased in the past few decades. It accounts for 35% to 40% of cases of gastric adenocarcinoma according to recent studies [2]. This increase can be explained by the recent changes in the pathological classification of GC [2,3]. Its etiology, pathological and clinical characteristics, variable chemosensitivity, the tendency of metastasis, prognosis, and management remain incompletely understood. Due to its benign appearance and lack of specific symptoms, it usually presents at a late stage.

Small bowel volvulus (SBV) is a rare entity. It is categorized into primary (due to any congenital intestinal anomalies) and secondary types (due to any tumor, diverticulosis, pregnancy, postoperative adhesions, etc). Jejunal volvulus in particular is usually primary. We present a case of advanced gastric SRCC with peritoneal carcinomatosis presenting primarily as intermittent jejunal volvulus.

This case was accepted for poster presentation at the American College of Gastroenterology (ACG) 2021 conference. 

## Case presentation

**Figure 1:**
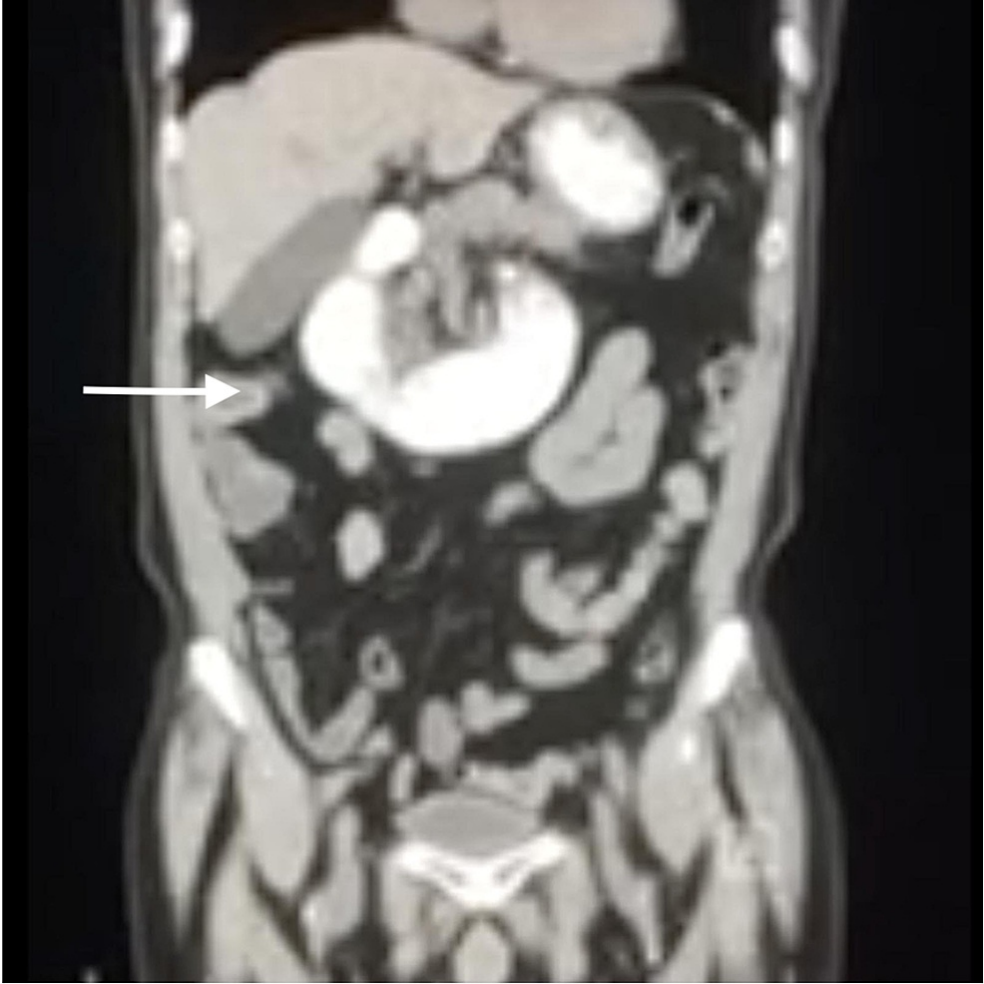
Coronal CT scan of abdomen and pelvis showing the abrupt swirled twisting of small bowel, also called the “coffee bean sign” CT, computed tomography

An 80-year-old Asian male presented to the emergency department for syncope. He had a history of non-bloody, non-bilious, and postprandial vomiting for the past two years. The patient also endorsed new-onset abdominal fullness, early satiety, undocumented weight loss, constipation, and excessive flatulence for the past three weeks. The patient had undergone a computed tomography scan (CT) of the abdomen at another facility three weeks prior to presentation. It showed a jejunal volvulus (shown in Figure 1). He was, then, treated conservatively and was later discharged home. The patient could not tolerate a liquid diet at home and experienced an episode of syncope after which he was brought to the hospital for further management.

**Figure 2:**
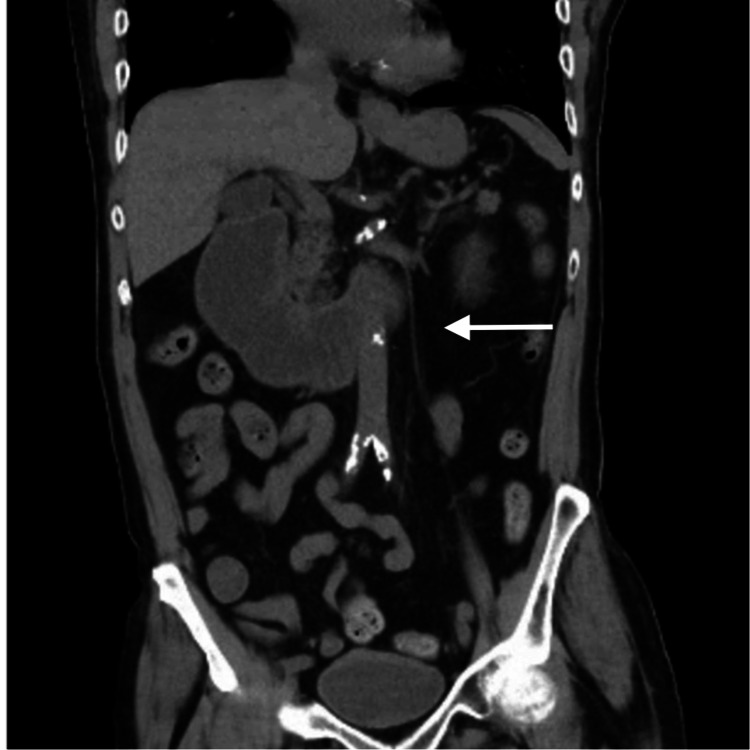
Coronal CT scan of abdomen and pelvis showing dilation of proximal small bowel up to 4.2 cm with a transition point at the superior mesenteric artery take-off CT, computed tomography

**Figure 3:**
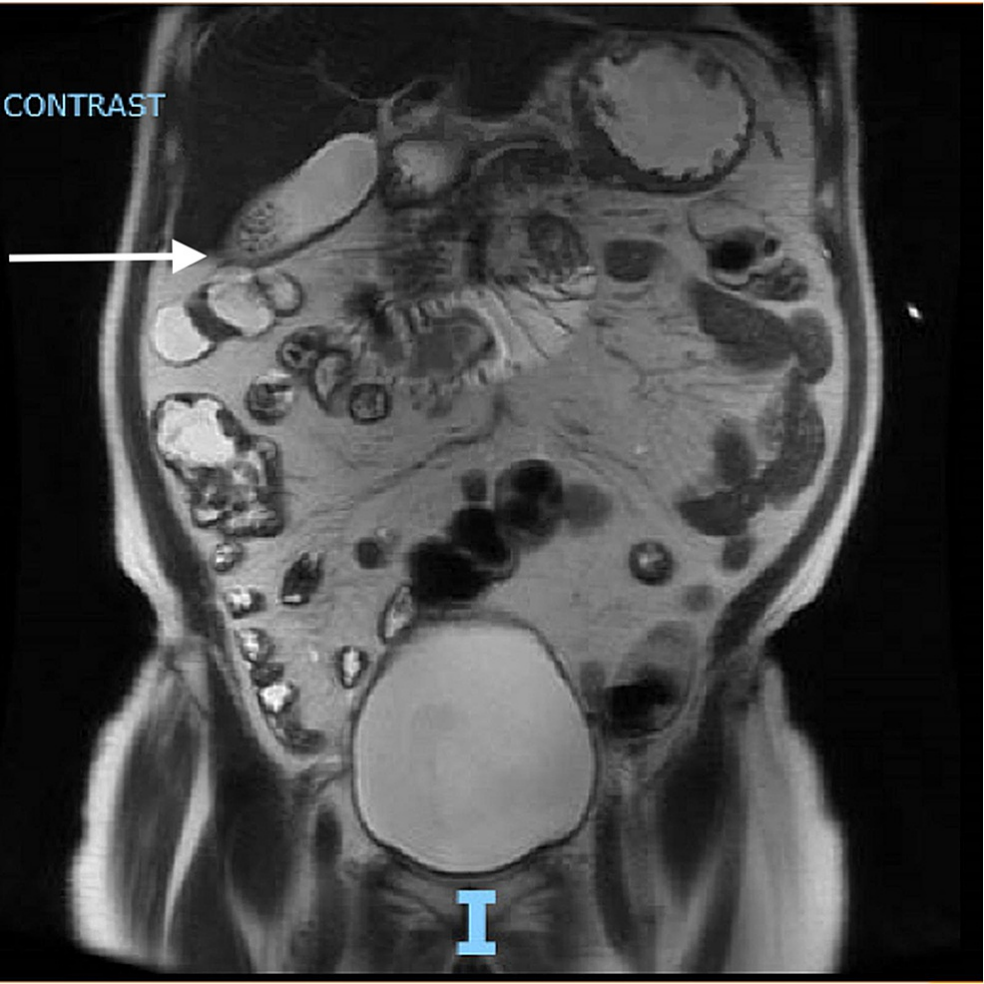
MRE of the abdomen and pelvis with intravenous and oral contrast showing duodenum distension of 3.4 cm; however, proximal jejunum never appeared fluid distended MRE, magnetic resonance enterography

A CT of the abdomen without contrast showed dilation of proximal small bowel up to 4.2 cm with a transition point at the superior mesenteric artery take-off (shown in Figure 2). It did not show any evidence of free air or ascites. Subsequent magnetic resonance enterography of the abdomen and pelvis with intravenous and oral contrast showed duodenum distension of 3.4 cm; however, proximal jejunum never appeared fluidly distended and a jejunal obstructing process could not be excluded (shown in Figure 3). No gastric lesions or thickening was appreciated on all imaging.

**Figure 4:**
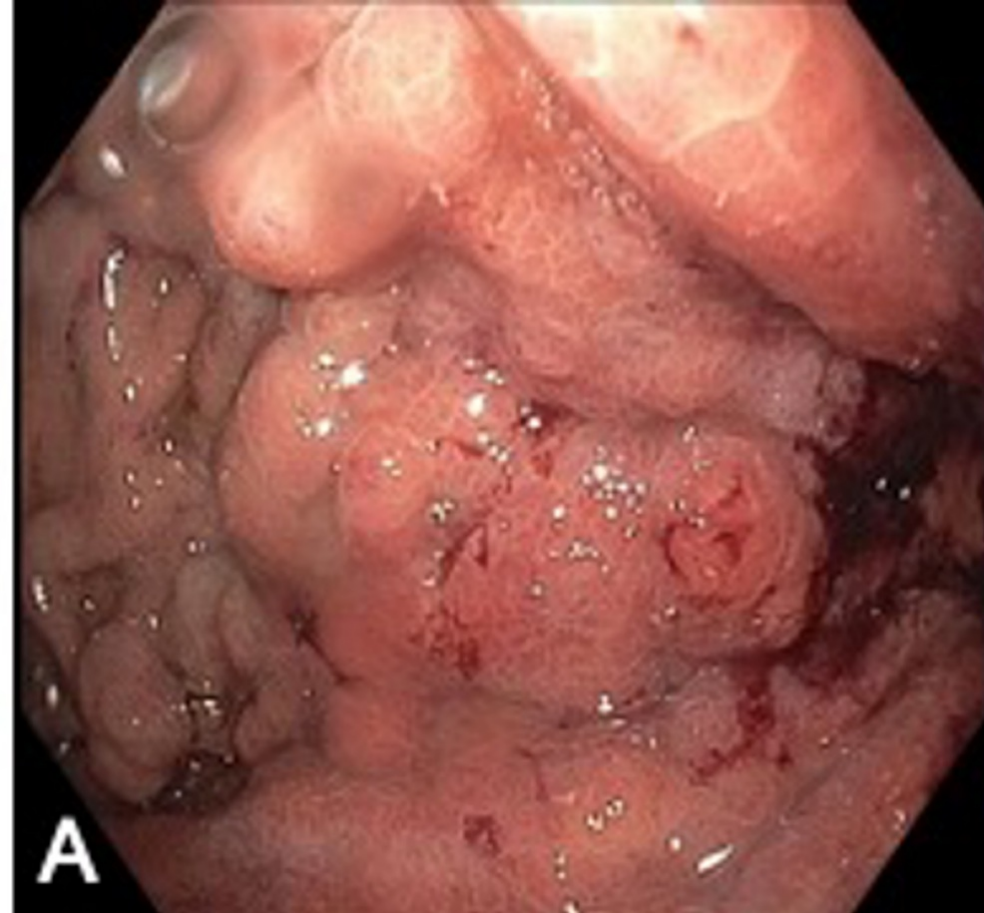
Deep enteroscopy showing multiple polyps ranging in size from 3 to 10 millimeters (mm) in the stomach body

**Figure 5:**
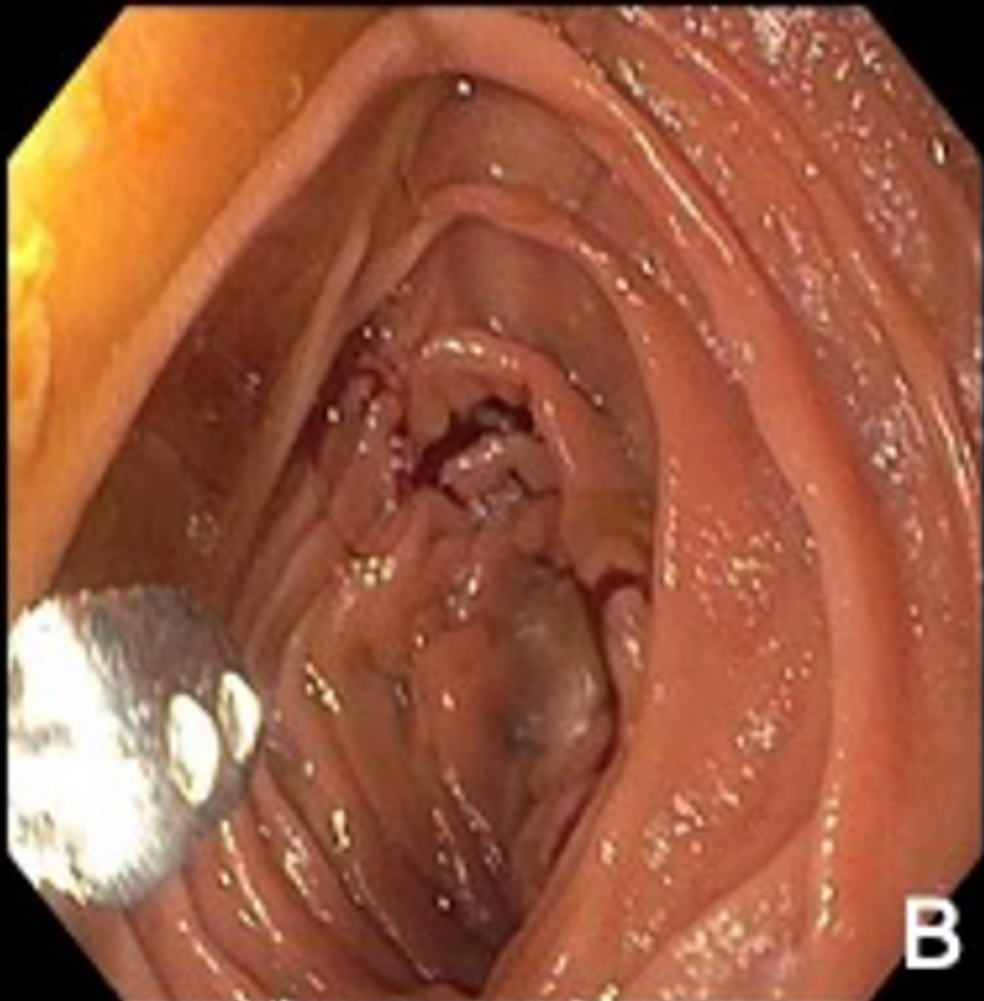
Deep enteroscopy showing antral circumferential mucosal thickening with mucosal changes under white light and narrow band imaging suggestive of carcinoma

Deep enteroscopy revealed antral circumferential mucosal thickening with mucosal changes under white light and narrow band imaging suggestive of carcinoma. Multiple polyps ranging in size from 3 to 10 millimeters (mm) were found in the stomach body (shown in Figures 4, 5). Dilated duodenum with transition area and obstructed proximal jejunum were noted with no intrinsic lesion or mucosal abnormalities. Multiple cold forceps biopsies were performed from gastric mucosa and jejunum. Histopathology from gastric mucosa revealed gastric adenocarcinoma, signet cell type. Proximal jejunum biopsy did not show significant diagnostic abnormalities.

The patient underwent diagnostic laparoscopy. It showed peritoneal implants throughout the abdominal cavity. A large, hard, and fixed mass was found in the distal stomach causing tethering of the distal duodenum. The frozen-section biopsy was positive for adenocarcinoma. Fashioned side-to-side loop duodenojejunostomy and partial omentectomy were done.

Pathology from the right upper quadrant peritoneal implant, peritoneal biopsy, and omentum showed metastatic foci ranging in appearance from gland forming to areas with signet ring cell features. The appearance was consistent with metastatic adenocarcinoma from the gastric primary site. It also showed human epidermal growth factor receptor 2 (HER2) negative, mismatch repair genes (MMR) proficient, and PD-L1CPS score of 5. The immunohistochemical stain was positive for hMLH1, hMSH2, hMSH6, and PMS2. The TNM (tumor, lymph nodes, metastasis) stage was M1, American Joint Committee on Cancer stage was IV. The level of serum tumor biomarker, carcinoembryonic antigen was 2.4 nanograms/milliliter (ng/mL).

The final diagnosis of stage IV gastric adenocarcinoma, signet ring cell type, poorly differentiated with peritoneal carcinomatosis was made. Palliative chemotherapy and palliative trans-pyloric duodenal stent were performed. After discussing the grave prognosis with the family, the family elected for hospice and comfort care only and the patient eventually expired within two months of initial diagnosis.

## Discussion

Gastric SRCC is a poorly differentiated, non-cohesive, and increasingly prevalent form of gastric adenocarcinoma. It frequently occurs in females and younger age groups as compared to other histological subtypes of GC. It could be explained by the presence of estrogen receptors on the gastric SRCC [4]. However, to date, the predilection to younger and female populations remains poorly understood [5].

It does not share the same risk factors as gastric non-SRCC. The role of *Helicobacter pylori* infection is controversial and the roles of cigarette smoking, obesity, and autoimmune gastritis are not well studied in SRCC [2]. It has two distinct forms. It can present as either early or advanced GC. SRCC in early GC (involves submucosa, irrespective of lymph node involvement) usually involves the middle part of the stomach. SRCC in advanced GC (muscle layer invasion) mostly occurs in the antro-pyloric region of the stomach.

SRCC does not exhibit the typical preneoplastic stages as seen in other GC subtypes [2]. It follows a specific oncogenesis pattern. Mutations in epithelial cadherin, coded by the CDH1 gene, plays a role in the initiation and progression of gastric SRCC [3]. Its deficiency causes loss of cell-to-cell adhesion, invasion to surrounding tissues, and accumulation of mucin in vacuoles. Mutations in Snail, Slug, Twist, and transformation growth factor B also play a significant role in pathogenesis [3].

Screening is not commonly practiced for GC except in endemic areas. Lack of specific symptoms often delays diagnosis and management and leads to dissemination of the disease process. Gastric SRCC predominantly metastasizes via peritoneum causing peritoneal carcinomatosis. The rate of peritoneal dissemination and lymph node metastasis is higher in advanced gastric SRCC. The advanced type also has the tendency to invade the duodenum as we observed in our case [6]. Therefore, some authors recommend routine laparoscopic evaluation before initiation of treatment [2].

Its diagnosis is usually made on the basis of symptoms suspicious of malignancy like weight loss, anemia, abdominal discomfort, prolonged history of gastric ulcer, or with the help of endoscopy or CT scan findings. However, histopathologic examination of the biopsy specimen is almost always required. Gastric SRCC has variable chemosensitivity [3]. 5-Fluorouracil and platinum-based chemotherapy are commonly used. Early-stage gastric SRCC can be resected endoscopically, whereas gastrectomy with adjunct chemoradiation therapy or palliative treatment remains the management of choice for advanced-stage gastric SRCC. There is also a scarcity of data regarding the long-term survival of patients with gastric SRCC. Its prognostic factors are undetermined. SRCC in early GC has a good prognosis and has less lymph node metastasis. SRCC in advanced GC has a poor prognosis with more nodal and peritoneal involvement [2].

To the best of our knowledge, this is the first case of gastric SRCC presenting as intermittent SBV. Volvulus is the twisting of the bowel around its mesentery. It is classified as either primary or secondary. Primary volvulus occurs usually due to any congenital anomaly. Intestinal malrotation is the most common cause of it. Secondary volvulus occurs due to any tumor, diverticulosis, pregnancy, prior surgery, or chronic constipation. It is relatively common in the cecum and sigmoid colon. Volvulus of other parts of the gastrointestinal tract is relatively rare. SBV and particularly jejunal volvulus are usually primary. SBV is a rare cause of small bowel obstruction in adults [7].

We believe our case is also unique due to the secondary nature of jejunal volvulus. Due to SBV rarity, relatively little is known about its true incidence, epidemiology, and presentation. Older age, male sex, associated comorbidities, history of prior abdominal surgery, and presence of necrotic bowel loops are the predictors of increased mortality in secondary SBV [8].

Clinical presentation is nonspecific unless it leads to acute small bowel obstruction with or without signs of peritonitis. Hematemesis and hematochezia are the signs of ongoing bowel ischemia and necrosis. Except in acute cases, the diagnostic imaging modality is CT scan of the abdomen. Its sensitivity to diagnose SBV is almost 60% and specificity is 93% [9]. It shows the pathognomic “whirlpool sign and peacock sign” for midgut volvulus. The whirlpool sign denotes the twisting of blood vessels around the base of mesentery [10]. The peacock sign denotes the twisting of the bowel around its mesenteric axis. A distended and fluid-filled proximal small bowel with an abrupt transition point also indicates SBV [11]. Additionally, angiography or multidetector CT improves diagnostic accuracy. Early surgical intervention is necessary to prevent bowel and vascular compromise. It is a reversible pathology if diagnosed and treated in a timely fashion. It was believed that tethering of distal duodenum by gastric SRCC caused intermittent jejunal volvulus leading to intermittent small bowel obstruction in our case.

## Conclusions

In conclusion, our case highlighted a rare subtype of gastric cancer, SRCC. The relative scarcity of data about its etiology, incidence, pathophysiology, treatment, and prognosis demands further studies to help in the timely diagnosis and management of this uncommon gastric cancer type. SBVs are usually primary in nature but can be secondary to tumors, diverticulosis, pregnancy, or post-surgical adhesions. This is the first case of secondary jejunal SBV unveiling advanced gastric SRCC without appreciable gastric mass on imaging.
